# Fulminant Myocarditis Following Gastric Bypass Surgery: A Case Report

**DOI:** 10.7759/cureus.102256

**Published:** 2026-01-25

**Authors:** Lela Kopaleishvili, Ivditi Okuashvili, Naia Amiranashvili, Nani Gonjilashvili, Mariam Lomidze, Tamaz Kheladze

**Affiliations:** 1 Cardiology, G. Chapidze Emergency Cardiology Center, Ivane Javakhishvili Tbilisi State University, Tbilisi, GEO; 2 Cardiology, G. Chapidze Emergency Cardiology Center, Tbilisi, GEO; 3 Internal Medicine/Cardiology, G. Chapidze Emergency Cardiology Center, Tbilisi, GEO; 4 Internal Medicine/Pulmonology, G. Chapidze Emergency Cardiology Center, Tbilisi, GEO; 5 Cardiac Surgery/Cardiology, G. Chapidze Emergency Cardiology Center, Tbilisi, GEO; 6 Emergency/Cardiology, G. Chapidze Emergency Cardiology Center, Tbilisi, GEO

**Keywords:** bariatric surgery, cardiogenic shock, fulminant myocarditis, micronutrient deficiency, roux-en-y gastric bypass

## Abstract

Fulminant myocarditis (FM) is a rare, life-threatening inflammatory disease of the myocardium characterized by sudden, severe cardiac dysfunction and hemodynamic instability. It often necessitates high-dose inotropic support and mechanical circulatory assistance, with a high risk of short-term mortality. We describe a 23-year-old woman who developed FM 18 months after undergoing Roux-en-Y gastric bypass. She presented with cardiogenic shock and ventricular arrhythmias precipitated by a period of clinical non-adherence and discontinued nutritional supplementation. Infectious and autoimmune workups were negative, but profound micronutrient and trace element deficiencies were identified. Emergency management included mechanical ventilation, inotropes, immunomodulatory therapy, and aggressive nutritional repletion. Cardiac magnetic resonance imaging confirmed myocarditis. Over two months, her left ventricular ejection fraction improved from approximately 25% to 56%, accompanied by full clinical recovery. This case illustrates the potential role of severe post-bariatric malnutrition in triggering myocardial inflammation and cardiovascular collapse. It underscores the importance of early recognition, multidisciplinary management, including immunomodulatory and nutritional therapy, and the necessity of vigilant long-term follow-up in patients after bariatric surgery to prevent such catastrophic outcomes.

## Introduction

Fulminant myocarditis (FM) is an uncommon but severe form of acute myocarditis, characterized by sudden and profound impairment of cardiac function, often leading to cardiogenic shock and life-threatening arrhythmias [1]. Viral infections and autoimmune disorders are the most frequently reported triggers, but other less recognized factors may contribute [2,3]. Bariatric surgery, particularly Roux-en-Y gastric bypass (RYGB), is a well-established intervention for morbid obesity that induces substantial weight loss; however, it is associated with nutrient malabsorption and requires lifelong supplementation [4,5]. Micronutrients such as thiamine (B_1_) and selenium are vital for myocardial energy metabolism and redox balance, and deficiencies can result in potentially reversible cardiac dysfunction. After bariatric surgery, failure to maintain adequate long-term supplementation may pose an underrecognized risk for serious cardiac complications. Although post-bariatric nutritional deficiencies are well documented, FM following bariatric surgery is exceedingly rare [6,7]. Here, we present a case of FM in a young woman 18 months after RYGB, in the context of severe post-surgical micronutrient deficiencies, highlighting the potential role of malnutrition in precipitating life-threatening cardiac events.

## Case presentation

A 23-year-old woman with a history of class III obesity (initial BMI 48 kg/m^2^) underwent an uncomplicated RYGB. While she achieved a total weight loss of 75 kg over the 18 months following surgery, she discontinued clinical follow-up and was non-adherent to the recommended multivitamin regimen. Approximately 18 months postoperatively, she was brought to the emergency department (ED) after a sudden syncopal episode at home, arriving in profound cardiogenic shock. The patient did not report any prodromal symptoms prior to the acute syncopal episode.

On presentation, the patient was unresponsive (Glasgow Coma Scale 6) and exhibited signs of severe hemodynamic compromise: blood pressure 70/40 mmHg, heart rate 150 bpm, respiratory rate 28/min, and oxygen saturation 85% on a non-rebreather mask. Jugular venous distension and diffuse pulmonary crackles were noted. She was emergently intubated and placed on mechanical ventilation, while intravenous norepinephrine and dopamine were initiated for hemodynamic support.

Bedside transthoracic echocardiography (TTE) revealed a severely reduced left ventricular ejection fraction (LVEF) of approximately 25% with global hypokinesia and mild mitral regurgitation. Coronary angiography was performed to rule out obstructive disease and demonstrated normal coronary arteries. Laboratory testing showed markedly elevated high-sensitivity cardiac troponin T (hs-cTnT - 0.104 ng/mL), N-terminal pro-B-type natriuretic peptide (NT-proBNP >35,000 pg/mL), and inflammatory markers, including C-reactive protein (CRP) 176.6 mg/L and leukocytosis (19.27 × 10^9^/L) (Table 1). A comprehensive nutritional screening revealed profound micronutrient and trace element deficiencies, most notably severe B_1_ deficiency (7 µg/L) and selenium deficiency (35 µg/L), 25-hydroxyvitamin D 3.5 ng/mL, and iron 3 µmol/L. Interestingly, while most micronutrients were depleted, the elevated serum vitamin B_12_ (>2000 pg/mL) likely reflected acute hepatocellular stress during cardiogenic shock rather than adequate storage.

**Table 1:** Laboratory findings The findings demonstrate severe myocardial injury and cardiac dysfunction, as evidenced by markedly elevated high-sensitivity troponin T and NT-proBNP levels, accompanied by lactic acidosis consistent with tissue hypoperfusion. Inflammatory markers were significantly elevated, while procalcitonin was not suggestive of bacterial sepsis. Nutritional assessment revealed profound deficiencies in vitamin D, iron stores, thiamine, and selenium, with paradoxically elevated vitamin B_12_ levels. Transaminase levels were within the normal range, and serum albumin remained within normal limits. NT-proBNP: N-terminal pro-B-type natriuretic peptide; ESR: erythrocyte sedimentation rate; AST: aspartate aminotransferase; ALT: alanine aminotransferase

Parameter	Result	Reference Range
Cardiac markers		
High-sensitivity troponin T	0.104 ng/mL	<0.014 ng/mL
NT-proBNP	>35,000 pg/mL	<125 pg/mL
Lactate	4.8 mmol/L	0.5-2.0 mmol/L
Inflammatory markers		
C-reactive protein	176.6 mg/L	<5 mg/L
Leukocyte count	19.27 × 10^9^/L	4-10 × 10^9^/L
ESR	46 mm/h	<20 mm/h
Procalcitonin	0.13 µg/L	<0.5 µg/L
Arterial blood gas		
pH	7.30	7.35-7.45
Lactate	4.8 mmol/L	0.5-2.0 mmol/L
Liver function tests		
AST	31 IU/L	<40 IU/L
ALT	32 IU/L	<41 IU/L
Nutritional parameters		
25-hydroxyvitamin D	3.5 ng/mL	>30 ng/mL
Serum iron	3.0 µmol/L	6.6-29.5 µmol/L
Transferrin saturation	2%	20-50%
Ferritin	8 ng/mL	15-150 ng/mL
Serum albumin	3.8 g/dL	3.5-5.5 g/dL
Vitamin B_12_	>2000 pg/mL	200-900 pg/mL
Electrolytes		
Sodium	133 mmol/L	135-145 mmol/L
Potassium	3.3 mmol/L	3.5-5.0 mmol/L
Magnesium	0.64 mmol/L	0.7-1.0 mmol/L
Micronutrient send-outs		
Thiamine (B_1_)	7 µg/L	>9 µg/L
Selenium	35 µg/L	70-150 µg/L

The patient’s admission laboratory evaluation revealed evidence of acute myocardial injury, systemic inflammation, metabolic stress, and severe micronutrient deficiencies, consistent with FM (Table 1).

**Figure 1:**
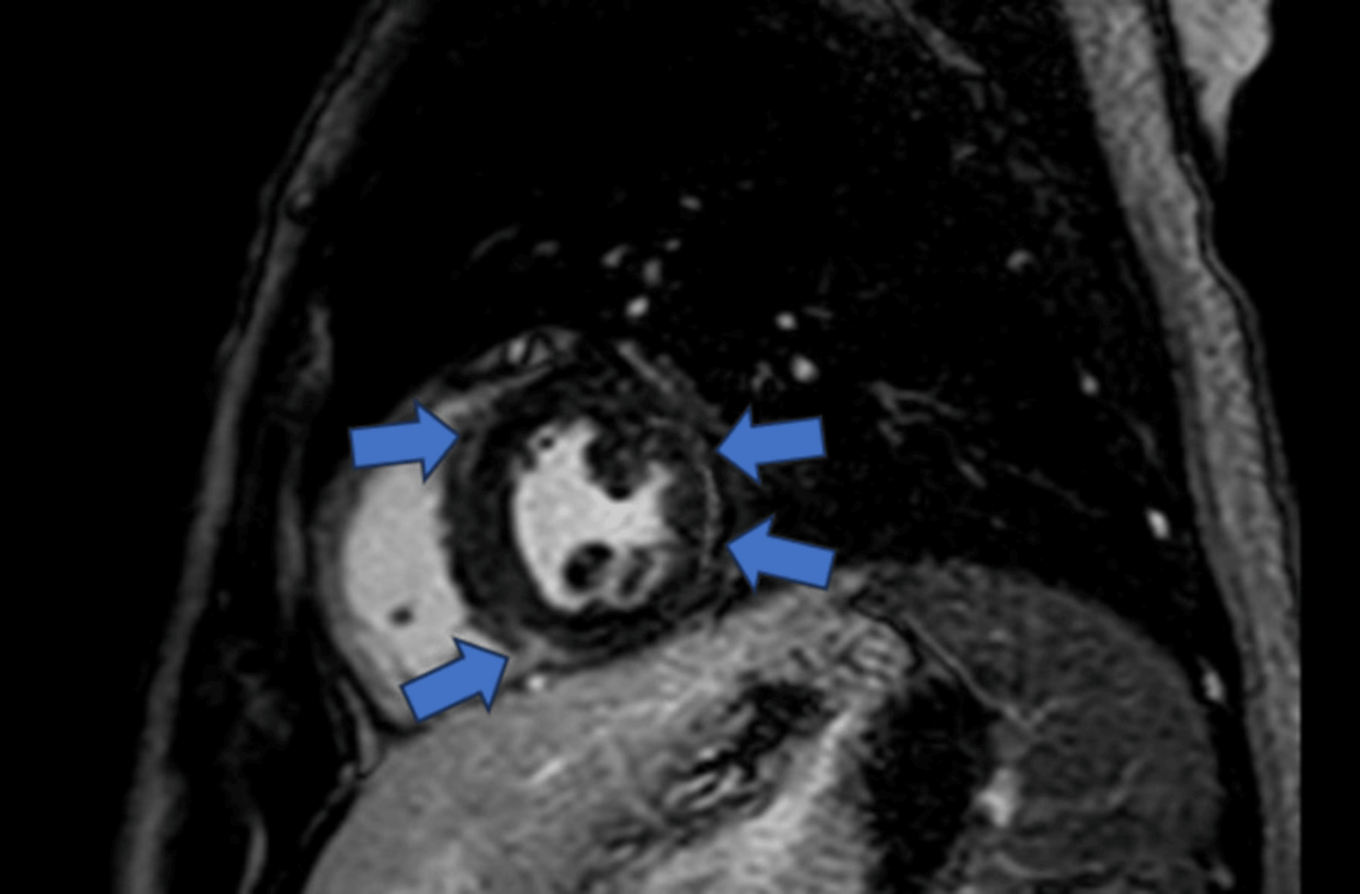
Cardiac Magnetic Resonance Imaging (CMR) Short-axis view demonstrating diffuse subepicardial late gadolinium enhancement (LGE) involving the lateral wall of the left ventricle (blue arrows), consistent with active inflammatory myocarditis. The imaging findings fulfill the Lake Louise criteria for the diagnosis of acute myocarditis [8,9].

On hospital day 10, cardiac magnetic resonance (CMR) imaging was performed. The findings met the Lake Louise criteria for myocarditis, demonstrating diffuse subepicardial late gadolinium enhancement (LGE) and myocardial edema (Figure 1) [8,9]. An extensive workup for viral (polymerase chain reaction (PCR)), toxic, and autoimmune etiologies yielded negative results.

Management was multifaceted, addressing both inflammatory and nutritional contributors to disease. Given the fulminant course, including mechanical ventilation and inotropic support, the patient received intravenous pulse methylprednisolone, which was subsequently transitioned to oral prednisone at 1 mg/kg/day with a gradual taper guided by hemodynamic and echocardiographic recovery.

Targeted micronutrient repletion was instituted in the context of documented deficiencies. B_1_ was administered intravenously at 500 mg twice daily for five days, then 250 mg IV daily for five days, followed by oral B_1_ 100 mg three times daily. Selenium supplementation was provided as oral 200 mcg daily, followed by 100 mcg daily as levels improved. Magnesium was replaced according to serum levels, and cholecalciferol supplementation was administered per standard protocols.

Rapid hemodynamic improvement was observed following initiation of high-dose parenteral nutritional repletion, allowing progressive weaning of cardiovascular support: inotropes (dobutamine) were discontinued by day 5, the norepinephrine dose was halved by day 3 and completely stopped by day 5, concomitant with normalization of serum lactate (from 4.8 to 1.6 mmol/L). The patient was successfully extubated on day 6, reflecting the swift reversal of metabolic and cardiovascular compromise primarily driven by targeted nutritional therapy. Follow-up echocardiography on day 10 demonstrated a partial recovery of the LVEF to 40%. The patient was discharged on a standard heart failure regimen and a rigorous nutritional supplementation program. At the two-month follow-up, she remained asymptomatic, with full recovery of LVEF (from ~20% at presentation to 56%).

## Discussion

This case highlights an unusual instance of FM following RYGB, where severe micronutrient deficiencies likely served as the primary driver for myocardial inflammation and cardiac failure [1,4,5]. The striking improvement after targeted nutritional therapy underscores the reversible nature of myocarditis induced by nutritional deficiency [10,11].

Post-bariatric malnutrition, particularly deficiencies in B_1_, selenium, vitamin D (3.5 ng/mL), and iron (3.0 µmol/L), has profound effects on cardiac metabolism and oxidative balance [4,5,10-14]. B_1_ is an essential cofactor for pyruvate dehydrogenase in myocardial energy metabolism; its depletion (7 µg/L) leads to impaired adenosine triphosphate (ATP) production and lactic acidosis, resulting in the acute circulatory collapse known as Shoshin beriberi [8,9]. Similarly, selenium is vital for maintaining redox balance via glutathione peroxidase; its deficiency (35 µg/L) increases oxidative stress within myocytes, potentially triggering an inflammatory response resembling Keshan disease [13,15]. Notably, the paradoxically elevated vitamin B_12_ level (>2000 pg/mL), in the setting of normal liver transaminases and preserved synthetic function on admission, may be influenced by acute systemic stress and cardiogenic shock, which can alter hepatic vitamin B_12_ handling and release. Consequently, elevated serum B_12_ should be interpreted cautiously and does not necessarily reflect adequate nutritional status. Serum albumin was within normal limits, consistent with preserved hepatic synthetic function. While contributory factors such as supplementation cannot be excluded, correction of B_1_ and selenium deficiencies led to rapid hemodynamic recovery, supporting their causal role [10,13].

Malnutrition also disrupts immune regulation and enhances systemic inflammation, as reflected by an elevated CRP of 176.6 mg/L and increasing myocardial susceptibility to injury [3,16,17]. Although viral PCRs were negative, it remains possible that transient viral exposure acted as a secondary trigger in an already compromised host [2,3]. The concept of “nutritional myocarditis” aligns with previous reports of reversible cardiomyopathy and myocarditis secondary to malnutrition after bariatric surgery [6,7]. The attribution of myocarditis primarily to nutritional deficiency is reinforced by a comprehensive negative workup for viral, autoimmune, and toxic causes, the presence of multiple profound micronutrient deficiencies, and the rapid clinical and echocardiographic recovery observed following targeted nutritional repletion - occurring prior to any substantial effect from immunosuppressive therapy. Management of FM requires early recognition, hemodynamic stabilization, and correction of precipitating factors [1,18]. A multidisciplinary team approach, combining intensive care, cardiology, and nutrition specialists, was critical to survival in this case.

This experience underscores that nutritional assessment should be an integral component of managing cardiogenic shock in post-bariatric patients [4,5]. Limited access to long-term postoperative nutritional monitoring and cardiac follow-up may exacerbate the risk of severe complications in post-bariatric patients, particularly in low-resource settings. Preventive strategies must focus on patient education and lifelong adherence to supplementation [4,5]. Routine monitoring of B_1_, selenium, vitamin D, and iron levels is essential in the first postoperative year and annually thereafter [5]. Clinicians must maintain a high index of suspicion for nutritional myocarditis in patients presenting with unexplained heart failure following bariatric surgery, particularly when a history of poor follow-up is present [4,5].

## Conclusions

FM after RYGB is an exceedingly rare but life-threatening complication. Severe micronutrient deficiencies in post-bariatric patients can precipitate acute myocardial failure and cardiogenic shock by impairing cardiac energy metabolism, redox balance, and immune regulation. Early recognition and aggressive management of nutritional deficiencies, alongside standard heart failure therapies and immunosuppressive treatment, were pivotal in our patient’s survival and recovery. Given the limited number of reported cases, this experience underscores the critical importance of lifelong nutritional surveillance and strict adherence to supplementation in patients following bariatric surgery.
